# “Fear Has Big Eyes”: Illness Perception, Fear of Recurrence, and Generalized Anxiety in Post-Treatment Thoracic Cancer Patients: A Serial Multiple Analysis

**DOI:** 10.3390/jcm15051797

**Published:** 2026-02-27

**Authors:** Dariusz Krok, Ewa Telka, Sebastian Binyamin Skalski-Bednarz

**Affiliations:** 1 Institute of Psychology, University of Opole, 45-040 Opole, Poland; 2 Department of Radiotherapy, Maria Sklodowska-Curie National Research Institute of Oncology, Gliwice Branch, 44-101 Gliwice, Poland; etelka@io.gliwice.pl; 3 Faculty of Philosophy and Education, Catholic University of Eichstätt-Ingolstadt, 85072 Eichstätt, Germany; sebastian.skalski@ku.de; 4 Institute of Psychology, Ignatianum University in Cracow, 31-501 Kraków, Poland

**Keywords:** thoracic cancer, illness perception, fear of recurrence, generalized anxiety, meaning-making, cognitive-motivational mediation

## Abstract

**Background/Objectives**: Although illness perception has been examined in oncology populations, there is a lack of empirical studies focusing specifically on post-treatment thoracic cancer patients and on the mechanisms through which illness perception relates to fear of cancer recurrence and generalized anxiety. In particular, prior research has rarely tested meaning-making and changes in beliefs and goals as mediating factors. This study aimed to examine the mediating roles of meaning-making and changes in beliefs and goals within a serial multiple mediation model between illness perception, fear of recurrence, and generalized anxiety. **Method**: A cross-sectional study was conducted with 284 thoracic cancer patients (149 men and 135 women) who had completed treatment. Participants completed validated self-report measures assessing illness perception, meaning-making, changes in beliefs and goals, fear of cancer recurrence, and generalized anxiety. Hierarchical regression analyses and serial multiple-mediation models based on path analysis were employed to examine direct and indirect associations among variables. **Results**: Negative illness perception was positively associated with fear of recurrence and generalized anxiety, while positive illness perception predicted lower levels of both outcomes. Path analyses revealed that meaning-making and changes in beliefs and goals jointly mediated the relationships between illness perceptions and psychological distress. Specifically, adaptive meaning-making and belief–goal restructuring were associated with lower fear of recurrence and generalized anxiety, whereas maladaptive forms were associated with higher levels of both outcomes. **Conclusions**: Findings indicate that both negative and positive illness perceptions influence post-treatment emotional adjustment in thoracic cancer patients through mediation effects. Based on the meaning-making model, interventions targeting maladaptive illness perceptions, promoting meaning-making, and supporting adaptive changes in personal beliefs and goals may reduce fear of recurrence and anxiety. These results support the incorporation of meaning-centered strategies into psychosocial oncology care, emphasizing cognitive–motivational cognitive-motivational factors as critical targets for improving emotional well-being in cancer survivorship.

## 1. Introduction

Illness perception plays a critical role in shaping how patients with cancer understand and respond to their disease, as it influences cognitions, emotional reactions, and the ability to find meaning in the illness experience [1,2]. The way patients perceive their illness also guides their health behaviors and treatment adherence, making illness perceptions a key target for interventions aimed at improving patient well-being. Understanding and addressing maladaptive illness perceptions is therefore essential for reducing emotional burden and supporting psychological adjustment in patients with thoracic cancer. Empirical insights from this research inform practical guidelines and the development of targeted psychosocial interventions.

### 1.1. The Importance of Illness Perception in Cancer and Its Associations with Emotional Reactions

The prominent role of illness perception in cancer patients stems largely from the fact that they frequently reflect on their illness and actively analyze the changes that occur throughout its progression. This has several implications for patients’ day-to-day coping with the illness. Patients with more positive or realistic perceptions are more likely to engage in adaptive coping, whereas negative or catastrophic perceptions may lead to avoidance, denial, or maladaptive coping [3,4]. Research has demonstrated that negative perceptions of cancer in the form of threatening, uncontrollable, or disruptive thoughts are associated with higher levels of negative affect in patients with different types of cancer [5], a poorer health-related quality of life among thyroid cancer survivors [6], and higher levels of fear and depression symptoms in gastrointestinal cancer patients [7]. In contrast, positive illness perceptions serve as protective and salutary factors, being associated with better quality of life conceptualized within functional domains (i.e., physical, role, cognitive, emotional, and social functioning) and lower perceived illness severity in patients with breast cancer [8], resilience in patients with different cancers [2], and psychosocial adaptation in young and middle-aged kidney transplant recipients [9].

The bipolar approach to illness perception is grounded in theoretical and empirical research indicating that positive and negative illness perceptions are independent, yet complementary dimensions. According to Janowski et al. [10], it is possible to distinguish commonly occurring meaning categories that individuals attribute to their illness: challenge, enemy, punishment, weakness, relief, benefit, loss, or value. According to these authors, these categories can be grouped into positive and negative domains—an approach we adopted analogously in our study.

Similarly, in the framework proposed by Broadbent et al. [11], eight independent dimensions constituting the broader construct of illness perception were identified. These dimensions can be categorized as positive (e.g., perceived personal control, illness coherence) or negative (e.g., concern about the illness, emotional response). Empirical studies have confirmed the validity of this approach [10,12]. Analyzing positive and negative dimensions separately allows for capturing the unique contributions of adaptive and maladaptive illness-related beliefs and their differential associations with fear of cancer recurrence, anxiety, and meaning-making processes.

Although the previous findings provide a coherent picture of the relationships between illness perception and patients’ psychological factors and responses, two important points should be noted. First, prior studies have only to a limited extent examined positive and negative illness perceptions simultaneously as independent and complementary dimensions. Second, there remains little empirical research investigating the relationship between illness perceptions and emotional responses in patients with thoracic cancer, despite the high prevalence and mortality associated with these malignancies. In 2025, thoracic cancers—primarily lung, trachea, and bronchus—remained the leading cause of cancer-related mortality worldwide, claiming approximately 1.8 million lives annually and accounting for an estimated 2.5 million new cases each year, or roughly 12.4% of all new cancer diagnoses globally [13]. These data highlight the pressing need for research in this area.

Research on psychosocial resources, illness perception, and cancer-related worry in Chinese early-stage lung cancer patients following surgery demonstrated that higher symptom burden and more negative illness perceptions were associated with elevated levels of cancer-related worry [14]. These findings suggest that, even after successful tumor removal, patients may continue to experience psychological and physical challenges during recovery, highlighting the importance of addressing both emotional and cognitive aspects of post-surgical adjustment. The findings also indicated differences in the associations between various patterns of illness perception and psychological and physical symptoms in patients with newly diagnosed advanced non-small cell lung cancer [1]. Among the participants, three patterns were identified: (1) a “struggling” profile that showed the most negative illness perceptions, (2) a “coping” profile that reflected generally positive perceptions, and (3) a “coping but concerned” profile that included elevated illness-related concerns but otherwise maintained relatively positive perceptions. The results indicated that the “struggling” profile, which included the largest subgroup, was associated with the highest symptom severity, the lowest levels of perceived personal control, and the highest levels of negative emotional responses and concerns. Mean anxiety and depression scores in this group were significantly higher than in the other profiles, with 28% and 35% of patients, respectively, reporting moderate to severe anxiety and depressive symptoms [1]. These findings suggest that the impact of negative illness perception may differ from that of positive illness perception not only in magnitude but also in nature.

In conclusion, the available evidence indicates that negative illness perceptions have a qualitatively and quantitatively different impact on patient outcomes than positive perceptions. Whereas positive perceptions may facilitate adaptive coping and resilience, negative perceptions are associated with heightened emotional distress and greater physical symptom burden, highlighting the importance of examining the specific associations of illness perceptions with fear of cancer recurrence and generalized anxiety.

### 1.2. Meaning-Making and Changes in Beliefs and Goals as Mediating Factors

There is empirical evidence suggesting that cognitive–motivational factors, such as meaning-making and changes in beliefs and goals, may mediate the relationship between illness perception and fear of recurrence and anxiety. Meaning-making is understood as a cognitive, continuous process in which individuals interpret experiences, events, and information, attributing personal significance by linking them to their existing knowledge, beliefs, and values [15,16]. As a consequence, people can construct a coherent and comprehensible understanding of themselves and the world. Research has demonstrated that meaning-making plays a vital role in the adjustment of cancer patients, promoting positive adjustment to the illness and posttraumatic growth [17], enhancing coping with stress [18], and reducing anxiety and depressive symptoms [19]. By facilitating a coherent understanding of the illness and its impact, meaning-making serves as a key psychosocial resource that supports psychological adjustment and overall emotional well-being throughout the cancer trajectory.

Meaning-making was found to play a mediating role in psychological processes among cancer patients. In a sample of Chinese patients with various types of cancer, meaning-making mediated the relationship between illness perception conceptualized as discrepancies with personal beliefs and posttraumatic growth [20]. Meaning-making was also a mediator between perceived and received social support and illness acceptance among Polish breast cancer patients [21]. In a longitudinal study, personal meaning (i.e., a related form of meaning-making) mediated the effect of meaning-centered group psychotherapy for cancer survivors on depressive symptoms [22]. However, some studies have yielded inconclusive or mixed results. Among patients with advanced lung and prostate cancer, path analyses showed that meaning-making did not mediate the relationship between a specific form of illness perception—perceived injustice (i.e., appraisals of one’s illness as unfair, severe, and irreparable)—and psycho-spiritual outcomes, including anxiety, depressive symptoms, anger about cancer, and anger toward God [23]. This suggests that the mediating role of meaning-making may depend on additional factors or may be specific to particular emotional responses.

The mediating role of meaning-making is fully illuminated within the meaning-making model, which provides a comprehensive framework for understanding how meaning shapes human functioning in the context of traumatic experiences, such as chronic illness [24,25]. The meaning-making model explains how individuals cope with stressful events, such as trauma or illness, by reconciling their existing core beliefs (global meaning) with new, challenging situations (situational meaning) to reduce stress and find purpose. Within this framework, meaning-making facilitates the adjustment of one’s understanding of events or beliefs to restore a sense of coherence and meaning in life. The process of meaning making thus enables the integration or modification of existing beliefs and goals (i.e., meaning made). It is associated with the cognitive processing involved in evaluating one’s current life situation. Through interpreting ongoing events, individuals are likely to revise their understanding of the causes of a given event or re-evaluate their personal beliefs and life goals [26,27]. In this way, they develop a more benign or less threatening interpretation of their situation. In the context of serious illness, such as post-treatment cancer, this process can help patients reframe the disease experience, find purpose in life beyond the illness, and maintain engagement with meaningful activities, thereby mitigating the impact of fear, anxiety, and uncertainty on overall well-being.

Research confirms the theoretical validity of considering also changes in beliefs and goals—an important motivational factor—in the context of cancer. The concept of changes in beliefs and goals is rooted in the meaning-making model, which posits that individuals’ appraisals of events may challenge and modify their existing beliefs and goals [16,25]. As a result, the discrepancy that emerges between global meaning and situational meaning may negatively affect psychological adjustment. Changes in beliefs and goals refer to modifications in an individual’s core assumptions about the self, the world, and the future, as well as in personally valued life aims and priorities, typically occurring in response to significant or stressful life events (e.g., serious illness, trauma, or existential loss) [26]. Thus, this construct reflects the extent of cognitive-existential change through which an illness challenges previously held assumptions and life plans.

Among patients with various types of cancer, more positive changes in personal beliefs, social relationships, and current/future priorities were associated with more positive affect, whereas more negative changes were associated with more negative affect and less positive affect. In addition, positive and negative changes were unrelated [28]. Violating beliefs and goals (i.e., the presence of negative changes in one’s system of beliefs and goals) was positively associated with affective symptoms, including anxiety, depression, and irritability, and negatively associated with meaning in life and coping strategies [7]. A systematic review of the literature on life goals in cancer patients showed that experiencing cancer can negatively affect the pursuit of life goals, significantly impacting daily functioning and quality of life; however, in some cases (e.g., through positive reinterpretation of daily activities), it may also lead to beneficial changes in life goals [29]. Changes in specific illness-related beliefs (i.e., uncertainty in illness and cognitive avoidance) also mediated the association between treatment and fear of recurrence in women diagnosed with breast and gynecological cancer [30]. These findings underscore the central role of beliefs and goals in shaping psychological adjustment and emotional reactions among cancer patients.

Although prior research has reported moderate associations among these variables, no study, to our knowledge, has examined meaning-making and changes in beliefs and goals—key cognitive–motivational factors—within a serial multiple mediation framework, incorporating both serial and parallel effects, in the relationships between negative vs. positive illness perception and fear of recurrence and generalized anxiety in patients with thoracic cancer. Moreover, reliance on specific, heterogeneous cancer populations limits the applicability of these findings to the thoracic cancer population [15,20,23]. By focusing on this particular cancer type and employing a path analysis approach, the present study addresses an existing gap and advances understanding of the factors that may mitigate fear of recurrence and anxiety in thoracic cancer patients.

### 1.3. The Present Study

**Figure 1:**
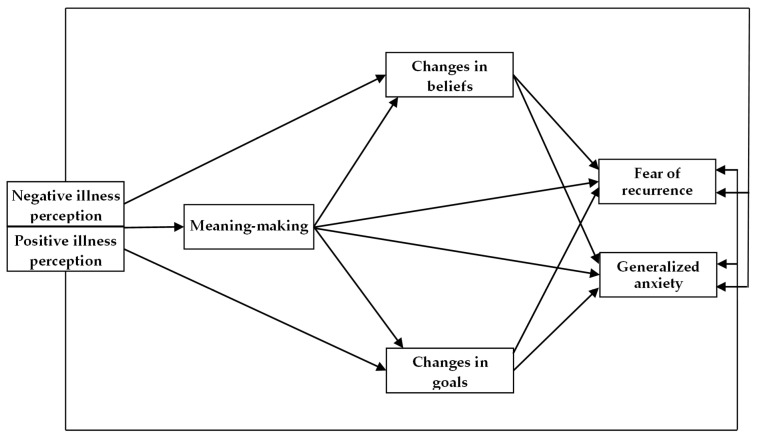
The theoretical model among the study variables.

The primary aim of the present study was to examine the mediating roles of meaning-making and changes in beliefs and goals within a serial multiple mediation model, in which negative and positive illness perception served as independent variables, and fear of recurrence and generalized anxiety as dependent variables, among patients with thoracic cancer who underwent treatment (see Figure 1). We also explored whether making changes in beliefs and goals acted as single mediators, highlighting their unique role in the experiences of cancer-related fear and anxiety among patients. Our study is novel in three ways: (1) it examined the direct and indirect relationships between illness perception and fear of recurrence and generalized anxiety in post-treatment cancer patients, (2) it scrutinized the mediational impact of cognitive–motivational factors (i.e., meaning-making and changes in beliefs and goals) on cancer patients’ emotional reactions, and (3) it included patients suffering from thoracic cancers, which are a major global health issue nowadays.

Grounded in the theoretical assumptions of the meaning-making model [24,25] and supported by prior empirical findings [5,17,26], we hypothesized that higher levels of negative illness perception would be associated with higher levels of fear of recurrence and generalized anxiety, as well as with lower levels of meaning-making and greater changes in beliefs and goals (H1a). Conversely, higher levels of positive illness perception were expected to be associated with the opposite pattern of associations with these variables (H1b). Furthermore, we hypothesized that meaning-making would act as a single mediator in the relationship between negative and positive illness perception and both distress outcomes (H2). Finally, we predicted that meaning-making and changes in beliefs and goals would function as serial and parallel mediators, jointly transmitting the effects of illness perception on fear of recurrence and generalized anxiety (H3).

## 2. Materials and Methods

### 2.1. Participants and Procedures

A total of 284 patients (149 men and 135 women) with an earlier diagnosis of thoracic cancer participated in the study. The sample comprised patients diagnosed with three main types of thoracic cancer: lung, bronchial carcinoma, and trachea, all of whom had received oncological treatment in specialized cancer units. The treatments administered included radiotherapy, chemotherapy, targeted therapy, and immunotherapy. Participants ranged in age from 33 to 85 years (M = 51.33, SD = 12.70). Regarding educational attainment, 24 participants (8.4%) had completed primary education, 62 (21.8%) had basic vocational education, 123 (43.4%) had secondary education, and 75 (26.4%) had higher education. Inclusion criteria were established to ensure patient safety, methodological rigor, ethical integrity, and the relevance and applicability of the study results. They were as follows: (1) a confirmed diagnosis of cancer at stages I to III; (2) absence of comorbid medical conditions that could substantially influence responses (e.g., stage IV cancer or severe cardiovascular disease); (3) sufficient cognitive capacity to complete self-report questionnaires; (4) a positive response to oncological treatment; (5) completion of active treatment with current remission status. These criteria ensured the methodological rigor and representativeness of the sample selection for the purposes of the present study.

### 2.2. Procedure

A purposive sampling method, commonly used in oncology research, was employed to ensure the inclusion of participants with specific characteristics and clinical criteria relevant to the study’s objectives. Participants were recruited during follow-up visits for their medical treatment in oncology units between December 2024 and November 2025. Potential participants were initially identified and approached by oncologists who assessed their medical status in accordance with the predefined inclusion criteria. Patients who met the study criteria were subsequently invited to participate and provided with detailed information about the study’s aims, procedures, and ethical safeguards. Written informed consent was obtained from all participants prior to their inclusion in the study. In total, 341 patients were invited to participate. Of these, 35 patients declined to participate, and 22 were excluded due to medical conditions that did not meet the eligibility criteria. As a result, the final study sample consisted of 284 participants, yielding a response rate of 83.3%.

Participation in the study was entirely voluntary and anonymous. All procedures were conducted in accordance with ethical standards, and participants were assured of the confidentiality of their responses and of their right to withdraw from the study at any time, without consequences for their medical care.

### 2.3. Measures

*Illness perception.* Illness perception was assessed using a short version of the Disease-Related Appraisals Scale [10], which assesses cognitive appraisals related to an individual’s illness. The 28-item scale comprises two subscales: negative illness perception, which measures negative appraisals of illness perceived in terms of threat, obstacle, and loss (16 items), and positive illness perception, which views the illness as a challenge and a source of value despite the difficulties experienced (12 items). The scale was developed within the stress and coping research paradigm [31], which enables the operationalization of a highly important construct in the context of illness: the primary appraisal of one’s own illness situation. Participants rated each item on a 5-point Likert scale ranging from 1 (strongly disagree) to 5 (strongly agree), with higher scores indicating greater negative or positive illness perception, respectively. In the current sample, internal consistency was high, with Cronbach’s alpha coefficients of 0.87 for negative illness perception and 0.85 for positive illness perception.

*Meaning-making.* Meaning-making was assessed using the Meaning-Making Questionnaire (MMQ) [32]. The aim of the MMQ is to measure an individual’s cognitive and reflective capacity to understand, interpret, and integrate challenging, stressful, or ambiguous life events into coherent systems of personal meaning, beliefs, and life goals. In the context of illness, this process reflects efforts to restore a sense of purpose and coherence following disruptive experiences. The questionnaire consists of 8 items rated on a 5-point Likert scale ranging from 1 (never) to 5 (very often). Higher total scores indicate a more frequent and active engagement in meaning-making processes. In the present study, the MMQ demonstrated good internal consistency, with a Cronbach’s alpha coefficient of 0.88.

*Changes in beliefs and goals.* Changes in beliefs and goals were assessed using the Scale of Changes in Beliefs and Goals [33]. This instrument evaluates the extent to which a patient experiences a serious illness as a disruption or violation of core personal beliefs and life goals. It captures the cognitive and motivational consequences of illness by examining how individuals reassess their worldview, self-concept, and future-oriented aspirations in response to the disease. The scale consists of 20 items and comprises two subscales: changes in beliefs, which reflect the degree to which illness has altered one’s beliefs about the self, others, and the world (10 items), and changes in goals, which assess the extent to which illness has interfered with important life goals (10 items). Items are rated on a 7-point Likert scale ranging from 1 (not at all) to 7 (very much), with higher scores indicating greater perceived disruption. In the present study, Cronbach’s alpha coefficients were 0.83 and 0.88 for changes in beliefs and goals, respectively.

*Fear of recurrence.* Fear of cancer recurrence was assessed using the 8-item Cancer Worry Scale (CWS) [34], which evaluates the frequency and intensity of concerns about the potential recurrence or progression of cancer, as well as the extent to which these worries interfere with everyday functioning. The items capture cognitive and emotional aspects of cancer-related worry, including intrusive thoughts, emotional distress, and behavioral consequences associated with fear of recurrence. Participants responded to each item using a 4-point Likert scale ranging from 1 (never) to 4 (almost always), with higher total scores indicating greater fear of cancer recurrence. Because the scale assesses worry about cancer recurrence in the same organ or the spread of disease to other parts of the body, it is suitable for use among post-treatment patients. In the present study, the scale demonstrated good internal consistency, with a Cronbach’s alpha coefficient of 0.83.

*Generalized anxiety.* Generalized anxiety was measured using the 7-item Generalized Anxiety Disorder Questionnaire (GAD-7) [35], a widely used self-report instrument designed to assess the overall severity of anxiety symptoms. The scale evaluates the frequency and intensity of common anxiety experiences over the past two weeks, including excessive worry, difficulty controlling worrying, restlessness, irritability, and fear of unexpected events. Participants rated each item on a 4-point Likert scale from 0 (not at all) to 3 (nearly every day), with higher total scores indicating greater generalized anxiety. The GAD-7 is suitable for both clinical and non-clinical populations and provides a brief, reliable measure of general anxiety symptoms. In the present study, the scale demonstrated good internal consistency, with a Cronbach’s alpha coefficient of 0.86.

### 2.4. Data Analysis

A priori power analysis conducted using G*Power 3.1 [36] for linear multiple regression (fixed model; R^2^ deviation from zero) indicated that a minimum sample size of 273 participants was required to achieve 80% statistical power (α = 0.05), assuming a small-to-moderate effect size (f^2^ = 0.05). To account for potential statistical errors and to ensure robust results, the final sample was increased to *N* = 284.

Given that all measures are self-reported, we conducted additional analyses to ensure construct distinctiveness. First, Harman’s single-factor test was applied to assess common method bias. An unrotated exploratory factor analysis revealed that the first factor accounted for 25.13% of the total variance, indicating that common method bias was not a significant concern in the present study [37]. We also confirmed that the distributions of all study variables were approximately normal, with skewness < 0.93 and kurtosis < 0.62. Next, the assessment of multicollinearity in the regression analysis indicated acceptable levels, with VIF values ranging from 1.46 to 1.64, suggesting no problematic multicollinearity. Furthermore, the correlation matrix indicated that the squared correlations (r^2^) among the study variables ranged from 0.0025 to 0.23, with the highest r^2^ observed between meaning-making and fear of recurrence (r^2^ = 0.23) and between fear of recurrence and generalized anxiety (r^2^ = 0.23). These values are below 0.50, suggesting that the constructs retain unique variance and are not excessively overlapping. Missing data were handled in AMOS using Full Information Maximum Likelihood (FIML), which estimates model parameters from all available data points without imputing missing values, providing unbiased estimates under the assumption that data are missing at random. Finally, bootstrapping with 5000 samples was applied to obtain 95% bias-corrected confidence intervals for estimating indirect effects [38].

Preliminary analyses of illness perception, meaning-making, changes in beliefs and goals, fear of recurrence, and generalized anxiety were based on Pearson correlation coefficients. To more precisely examine the relationships between predictors and each dependent variable, hierarchical regression analyses were conducted. Hierarchical regression allows predictors to be entered in theoretically or empirically determined blocks, examining incremental variance explained by conceptually ordered predictors. Finally, to test hypotheses regarding serial and parallel mediation, path analysis within the structural equation modeling (SEM) framework was conducted using maximum likelihood estimation in AMOS 26 software. Bootstrap resampling (5000 samples) with 95% confidence intervals was used to estimate indirect effects. Model fit was evaluated using the standard multiple indices: χ^2^/df (≤5), AGFI, NFI, CFI, TLI (≥0.90), RMSEA (≤0.08), SRMR (≤0.08), and Hoelter’s index (≥200) [39].

## 3. Results

### 3.1. Correlational Analysis and Hierarchical Regression

The first step in the statistical analysis was to calculate preliminary statistics for all study variables, which are presented in Table 1 (means, standard deviations, and Pearson correlations).

As hypothesized, the correlational results showed a coherent, theoretically consistent pattern across the study variables. Age was positively related to negative illness perception, changes in beliefs, and generalized anxiety, and negatively related to meaning-making. Negative illness perception was positively associated with maladaptive outcomes such as changes in beliefs, changes in goals, fear of recurrence, and generalized anxiety, with the strongest association observed for generalized anxiety. Simultaneously, negative illness perception was negatively related to positive illness perception and meaning-making. In contrast, positive illness perception was positively correlated with meaning-making and negatively related to changes in beliefs, changes in goals, fear of recurrence, and generalized anxiety, indicating its protective role against fear and distress.

Meaning-making was negatively associated with changes in beliefs, changes in goals, fear of recurrence, and generalized anxiety, underscoring its adaptive function in psychological adjustment. Changes in beliefs and goals were positively correlated, suggesting that cognitive and motivational reorganization often co-occur. At the same time, both variables were positively associated with fear of recurrence and generalized anxiety. Finally, fear of recurrence was positively correlated with generalized anxiety, reflecting a substantial overlap between cancer-specific fear and broader anxiety symptoms.

Following the correlation analysis, hierarchical regression was conducted to examine the extent to which illness perception, meaning-making, and changes in beliefs and goals (predictors) predicted fear of recurrence and generalized anxiety (dependent variables), respectively, while controlling for gender and age (covariates). The regression was conducted in four sequential steps. In the first step, covariates (gender and age) were entered. In the second step, negative and positive illness perceptions were added, followed by meaning-making in the third step. In the fourth step, changes in beliefs and goals were included. The results for fear of recurrence and generalized anxiety are presented in Table 2.

In the first hierarchical regression analysis, fear of recurrence was entered as the dependent variable. In the first step (Model 1), the overall regression equation was not statistically significant, *F*(2, 281) = 1.13; *p* = 0.332. In the second step (Model 2), the inclusion of negative illness perception and positive illness perception significantly improved the prediction of the dependent variable, fear of recurrence—the regression equation became statistically significant, *F*(4, 279) = 16.61; *p* < 0.001, and the explained variance increased by 18% (*p* < 0.001). In the third step (Model 3), adding the meaning-making dimensions further improved the prediction of the dependent variable, *F*(5, 278) = 35.18; *p* < 0.001, increasing the explained variance by 18% (*p* < 0.001). In the fourth step (Model 4), the inclusion of the dimensions of changes in beliefs and goals further improved the prediction of fear of recurrence, F(7, 276) = 29.25, *p* < 0.001, raising the explained variance by 6% (*p* < 0.01).

Since the aim of the analysis is test the incremental contribution of theoretically defined and ordered blocks of predictors, the final model (Model 4) is the statistically significant one that shows a meaningful increase in explained variance compared to the previous model with fewer predictors. Model 4, which included all predictors and covariates—Gender, Age, Negative Illness Perception, Positive Illness Perception, Meaning-Making, Changes in Beliefs, and Changes in Goals—provided the best overall prediction of fear of recurrence. Based on the significant regression coefficients, fear of recurrence will be lower when positive illness perception and meaning-making are higher and when changes in beliefs and goals are lower. The variables included in this model explained 43% of the variance.

In the second hierarchical regression analysis, generalized anxiety was entered as the dependent variable. The regression model in Step 1 (Model 1) was not statistically significant, *F*(2, 282) = 2.78, *p* = 0.064. Adding negative and positive illness perceptions in Model 2 (Model 2) significantly improved the prediction of generalized anxiety, *F*(4, 279) = 17.50, *p* < 0.001, accounting for an additional 18% of the variance. In Model 3, the inclusion of meaning-making further improved the model, *F*(5, 278) = 17.41, *p* < 0.001, increasing the explained variance by 4%. Model 4 introduced dimensions of changes in beliefs and goals, which significantly enhanced the prediction of generalized anxiety, *F*(7, 276) = 18.21, *p* < 0.001, accounting for an additional 8% of variance. Overall, the final model (Model 4) explained 32% of the variance in generalized anxiety. Significant predictors included negative illness perception, meaning-making, changes in beliefs, and changes in goals. Standardized regression coefficients indicated that higher levels of generalized anxiety were associated with lower presence of meaning-making, but with higher negative illness perception, and greater changes in beliefs and goals.

### 3.2. Serial Multiple Mediation Effects (Path Analysis)

To assess serial multiple mediation effects, we used path analysis within a structural equation modeling (SEM) framework. An initial model specifying directional paths among negative and positive illness perception (independent variables), meaning-making, changes in beliefs, changes in goals (serial and parallel mediators), fear of recurrence, and generalized anxiety (dependent variables) was first tested. However, the model including three mediators demonstrated an unsatisfactory fit to the data, χ^2^(3) = 79.70, *p* < 0.001; CMIN/DF = 26.56; AGFI = 0.30; NFI = 0.57; IFI = 0.58; CFI = 0.54; TLI = 0.41; RMSEA = 0.30; SRMR = 0.10; Hoelter’s index = 41. In addition, several paths in the initial model were not statistically significant (*p* > 0.05).

The model was subsequently refined using modification indices, theoretical plausibility, and parsimony, all of which were considered in the search for the optimal model [38,39]. The theoretical justification for the SEM model modifications was grounded in the meaning-making framework, which assumes that illness perception is associated with emotional outcomes (i.e., fear of recurrence and generalized anxiety) both directly and indirectly through meaning-making and subsequent changes in beliefs and goals [15,25]. During model refinement, several non-significant paths were identified. Their removal was not based solely on statistical criteria but was also guided by the theoretical assumptions of the meaning-making model. According to this framework, more constructive meaning-making is associated with fewer destabilizing changes in beliefs and goals, which, in turn, are linked to lower levels of fear of recurrence and generalized anxiety. Furthermore, indirect associations between illness perception and emotional outcomes, mediated by meaning-making and changes in beliefs and goals, are theoretically expected to be more prominent than direct effects.

**Figure 2:**
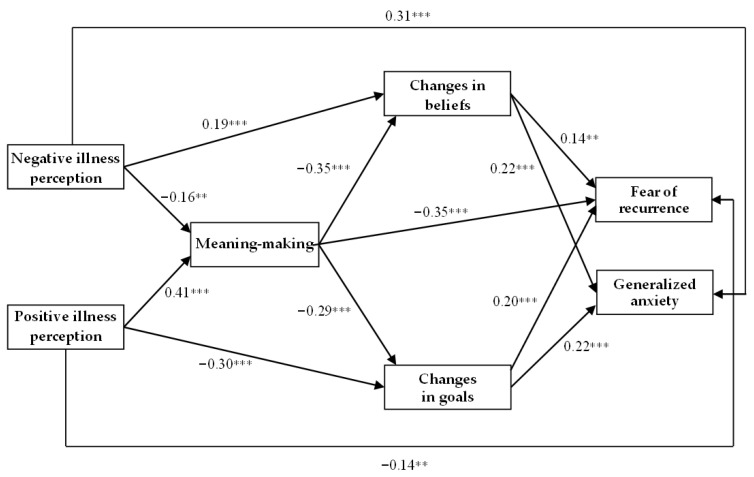
The final path analysis model between negative and positive illness perception, meaning-making, changes in beliefs and goals, fear of cancer recurrence, and generalized anxiety. ** *p* < 0.01, *** *p* < 0.001.

Based on these assumptions, we introduced modifications that primarily strengthened the sequential mediation process: illness perception → meaning-making → changes in beliefs and goals → fear of recurrence and generalized anxiety. This structure more closely aligns with discrepancy-reduction models of meaning-making, which posit that meaning-making operates through cognitive integration processes rather than exerting independent direct effects on emotional distress [16,24]. At the same time, selected direct paths from negative and positive illness perceptions to emotional outcomes were retained (i.e., negative illness perception → generalized anxiety; positive illness perception → fear of recurrence), as they are theoretically consistent with the meaning-making model. Specifically, the model suggests that threat appraisals may exert direct emotional effects, particularly when there is a strong discrepancy between individuals’ global beliefs and goals and their appraised meaning of the illness [25] (Figure 2).

The revised model showed a statistically substantial improvement in fit, χ^2^(5) = 9.58, *p* = 0.088; CMIN/DF = 1.91; AGFI = 0.95; NFI = 0.95; IFI = 0.97; CFI = 0.97; TLI = 0.91; RMSEA = 0.05; SRMR = 0.03; Hoelter’s index = 328. All fit indices fulfilled the required criteria for good model fit, indicating that the revised model more accurately captured the underlying associations among the variables examined in our study. In addition, a chi-square difference test was performed to compare the fit of the initial and final models. The result confirmed that the revised model provided a significantly better fit than the initial model, Δ*χ*^2^(2) = 70.12, *p* < 0.001. Taken together, these results indicate that the final model provides an optimal fit to the data. The final model is presented in Figure 2.

The results of the final path analysis model showed two direct effects: negative illness perception had a direct positive effect on generalized anxiety, whereas positive illness perception was directly and negatively associated with fear of recurrence. To test the proposed mediational mechanisms, indirect effects were examined using a bootstrapping procedure, which provides a reliable and robust method for estimation [39]. Table 2 presents the standardized bootstrapped estimates and 95% confidence intervals for the final mediation model, highlighting both serial and parallel mediation effects. Only effects whose empirical 95% confidence intervals do not overlap with zero are considered statistically significant. The results indicated four significant mediation effects operating in both serial and parallel forms, two effects operating only in parallel, and six single mediation effects (see Table 3).

The first two serial and parallel mediation models examined negative illness perception as the independent variable. In the first model, negative illness perception predicted higher levels of fear of recurrence through a sequential pathway involving meaning-making, changes in beliefs, and changes in goals. Specifically, higher levels of negative illness perception were associated with lower meaning-making, which, in turn, was linked to increased changes in beliefs and goals, ultimately resulting in higher fear of recurrence. In the second model, negative illness perception similarly predicted higher levels of generalized anxiety via the same sequential pathway. Higher negative illness perception was associated with weaker meaning-making, which was related to greater changes in beliefs and goals that, in turn, were associated with increased generalized anxiety. Mediation effects for fear of recurrence and generalized anxiety were comparable (E = 0.09, *p* < 0.001; E = 0.07, *p* < 0.001, respectively).

The next two serial and parallel mediation models focused on positive illness perception as the independent variable, and fear of recurrence and generalized anxiety as the dependent variables. The pattern of the relationship was similar in both cases. Positive illness perception predicted a lower level of fear of recurrence and generalized anxiety through a sequential pathway involving meaning-making and both changes in beliefs and changes in goals. Higher levels of positive illness perception were associated with more elaborate meaning-making, which was related to fewer changes in the domains of beliefs and goals, ultimately resulting in lower fear of recurrence and generalized anxiety. Comparison of standardized coefficients indicated a stronger mediation effect for fear of recurrence (E = −0.25; *p* < 0.001) than for generalized anxiety (E = −0.13; *p* < 0.001).

In addition to the serial multiple mediation models described above, two parallel mediation effects were also identified. Changes in beliefs and goals emerged as parallel mediators in the relationship of meaning-making with both fear of recurrence and generalized anxiety. Specifically, more expanded meaning-making was associated with fewer changes in beliefs and goals, which, in turn, was associated with a lower intensity of both fear of recurrence and generalized anxiety. Mediation effects for fear of recurrence and generalized anxiety were comparable (E = −0.11, *p* < 0.001; E = −0.14, *p* < 0.001, respectively). This finding implies that the mediating process operates with a similar strength in explaining both illness-specific distress (fear of recurrence) and more general emotional distress (generalized anxiety).

Finally, single mediation effects were found between illness perception and changes in beliefs and goals. Meaning-making mediated the relationship between negative and positive illness perception and changes in beliefs and goals. More precisely, higher negative illness perception was associated with lower meaning-making, which, in turn, was related to more unfavorable changes in beliefs and goals. In contrast, higher levels of positive illness perception were associated with a more expansive process of meaning-making, which, in turn, was linked to fewer changes in beliefs and goals. In both cases, the values of standardized coefficients were comparable for changes in beliefs and goals (E = 0.05, *p* < 0.01; E = 0.06, *p* < 0.01 for negative illness perception, and E = −0.14, *p* < 0.001; E = −0.12, *p* < 0.001 for positive illness perception), highlighting the central role of meaning-making in mitigating maladaptive changes in patients’ belief and goal systems. Meaning-making was also found to mediate the association of negative and positive illness perception with fear of recurrence, but not generalized anxiety. Specifically, higher negative illness perception was associated with lower meaning-making, which, in turn, was associated with greater fear of recurrence. Conversely, higher positive illness perception was associated with greater meaning-making, which, in turn, was associated with lower fear of recurrence.

## 4. Discussion

The present study examined the mediational function of meaning-making and changes in beliefs and goals in the relationship between negative and positive illness perception with fear of recurrence and generalized anxiety within a serial multiple mediation model in post-treatment thoracic cancer patients. To the best of our knowledge, this is the first study to apply a serial multiple mediation model including cognitive–motivational factors (i.e., meaning-making and changes in beliefs and goals) to examine the relationships between illness perception and fear and anxiety in patients with post-treatment thoracic cancer. Our hypotheses were fully or partially supported.

### 4.1. Associations Among Illness Perception, Fear of Recurrence, and Generalized Anxiety

Overall, the correlation and path analysis pattern supports a close association between negative and positive illness perception, fear of recurrence, and generalized anxiety, providing a strong empirical basis for subsequent mediation analyses. According to the first hypothesis (H1a), patients with higher levels of negative illness perception were expected to show higher levels of fear of recurrence and generalized anxiety, as well as lower levels of meaning-making and greater changes in beliefs and goals. Conversely, we anticipated an opposite pattern of associations for positive illness perception (H1b). The results fully confirm Hypotheses 1a and 1b, as negative illness perception was positively associated with fear of recurrence and generalized anxiety, whereas positive illness perception was negatively related to fear of recurrence and generalized anxiety.

These findings align with previous studies showing that negative perceptions of cancer are associated with stronger negative emotions and higher levels of fear and depression symptoms in patients with various types of cancer [5,7]. In contrast, positive illness perceptions have been linked to better psychosocial adaptation, such as among kidney transplant patients [10]. However, the present study advances existing knowledge—particularly regarding fear of recurrence—by demonstrating that the positive and negative facets of illness perception are inversely associated with fear of cancer recurrence. This is especially important given that fear of recurrence is a prevalent concern among cancer patients and refers to persistent worries about the possibility of cancer returning after treatment [40,41]. Such fears often intensify anxiety, compounding the emotional burden associated with the illness. In line with our findings, we suggest that patients with post-treatment thoracic cancer who perceive their illness as less threatening and disturbing, yet as offering both value and challenge, may approach recurrence-related fears with less apprehension and greater emotional resilience. This adaptive perspective can enable patients to engage more effectively in coping strategies, maintain a sense of control, and preserve daily functioning despite ongoing uncertainty. By fostering constructive cognitive and emotional processing, such appraisals may also facilitate meaning-making and promote the re-evaluation of personal goals and priorities. Consequently, these patients are better equipped to manage both fear of recurrence and generalized anxiety.

Moreover, considering the notable finding that the negative and positive dimensions of illness perception were relatively independent (r = −0.20), these results suggest that both diminishing perceptions of illness as threatening and life-disrupting and fostering perceptions of illness as a source of value and challenge may play complementary roles in supporting patients’ emotional functioning. By simultaneously reducing distress and strengthening emotional management, such balanced illness perceptions may help patients better manage anxiety, cope with future uncertainties, and engage more effectively with the challenges posed by their condition [14,27]. This highlights the potential importance of interventions that not only alleviate negative illness perceptions but also enhance positive, growth-oriented perceptions, thereby improving overall psychological adjustment [42].

### 4.2. The Serial Multiple Mediation of Meaning-Making and Changes in Beliefs and Goals

The key finding of our study demonstrates serial multiple mediational effects of meaning-making on beliefs and goals, revealing the complex, multidimensional role of cognitive–motivational factors in experiencing lower levels of anxiety and fear in post-treatment thoracic cancer patients. This indicates that how patients interpret and integrate their illness experiences influences subsequent adjustments in core beliefs and life goals, which, in turn, shape emotional responses to uncertainty and the threat of disease recurrence.

Hypothesis 2 assumed that meaning-making would act as a single mediator in the relationship between negative and positive illness perception, fear of recurrence, and anxiety. Our findings partially confirmed that meaning-making mediated the association of negative and positive illness perception with fear of recurrence, but not with generalized anxiety. Specifically, higher levels of negative illness perception were associated with reduced engagement in meaning-making, which, in turn, was linked to increased fear of recurrence. Conversely, higher levels of positive illness perception were associated with greater engagement in meaning-making, which, in turn, was associated with lower fear of recurrence. This suggests that the way in which cancer patients cognitively interpret and integrate their illness experience plays a significant role in shaping cancer-specific fears related to recurrence. Individuals who perceived their illness as more threatening or less meaningful were more likely to experience elevated fear of recurrence to the extent that they engaged in less constructive meaning-making, whereas more positive illness perceptions were linked to lower fear of recurrence through enhanced meaning-making processes. These findings are consistent with previous research highlighting the important role of meaning-making in the emotional reactions of cancer patients [17,21,23], suggesting that it functions as a key psychological mechanism through which illness perceptions shape patients’ emotional responses to the threat of cancer recurrence, either being related to distress when meaning-making is hindered or being associated with less fear when meaning-making is actively supported.

An interesting observation was that mediation was only found for fear of recurrence, but not generalized anxiety. This suggests that generalized anxiety appears to be influenced more directly by illness perceptions or by other mechanisms not captured by meaning-making alone, such as trait-level vulnerability, chronic worry, or intolerance of uncertainty. In that case, meaning-making may be particularly salient for illness-specific threat appraisals, rather than for more diffuse forms of psychological distress. Consistent with the meaning-making model [24,25], this distinction indicates that meaning-making appears to function as a key mechanism through which illness perceptions shape illness-specific fears, but may be less central to broader anxiety outcomes. As previous research indicates, fear of recurrence is more strongly associated with specific beliefs and thoughts concerning the current illness [41], whereas generalized anxiety relates to more general emotional responses [43]. Therefore, given the nature of cancer as an unpredictable and difficult-to-control disease, even patients who have successfully completed cancer treatment are more likely to interpret and construct illness-related experiences and feelings based on concrete and current concerns rather than general, trait-like emotional vulnerabilities.

Hypothesis 3 predicted that meaning-making and changes in beliefs and goals would function as serial and parallel mediators, jointly transmitting the effects of illness perception on fear of recurrence and generalized anxiety. This hypothesis was fully verified in the final path analysis model. More specifically, stronger negative illness perceptions were associated with reduced engagement in meaning-making, which was subsequently linked to greater changes in beliefs and goals, ultimately resulting in higher levels of fear of recurrence and generalized anxiety. In contrast, stronger positive illness perceptions were associated with more developed meaning-making, which was linked to fewer changes in beliefs and goals and, in turn, to lower levels of fear of recurrence and generalized anxiety. Specifically, the serial mediation pathway (illness perception → meaning-making → changes in beliefs and goals → emotional outcomes) indicates that cancer patients first engage in meaning-making efforts to interpret their illness experience, and that these efforts are subsequently associated with the extent to which their global beliefs and life goals are reorganized. When meaning-making is more constructive, it appears to reduce disruptive changes in beliefs and goals, which in turn are associated with lower levels of fear of recurrence and generalized anxiety. At the same time, the parallel mediation effects suggest that meaning-making and changes in beliefs and goals may also be independently related to emotional distress. This implies that cognitive integration of the illness experience and the restructuring of core beliefs and goals represent partially distinct, though interrelated, mechanisms of emotional adaptation.

These primary findings are in line with previous studies, which revealed a mediational role of meaning-making and changes in beliefs and goals between social support and illness acceptance among breast cancer patients [21], meaning-centered group psychotherapy and depressive symptoms in cancer survivors [22], and treatment and fear of recurrence in women diagnosed with breast and gynecological cancer [30]. This indicates that cognitive–motivational factors (i.e., meaning-making and changes in beliefs and goals) represent central elements through which illness perceptions influence emotional outcomes in patients with thoracic cancer.

However, the present study broadens our understanding of the emotional reactions of cancer patients in two key aspects: (1) it highlights the interplay of cognitive (i.e., meaning-making) and motivational (i.e., changes in beliefs and goals) factors in patients’ emotional responses, both at the level of illness-specific fear of recurrence and more generalized anxiety that is not necessarily related to the experienced illness, and (2) it provides empirical evidence for a dual pathway through which negative vs. positive illness perception exerts their influence on patients’ emotional reactions.

First, the interplay of cognitive and motivational factors, represented by sequential and parallel mediation pathways of meaning-making and changes in beliefs and goals, closely aligns with Park’s meaning-making model [15,24], which emphasizes that illness appraisals guide cognitive and motivational factors and ultimately psychological outcomes. Experiencing distress related to a negative perception of one’s illness motivates cancer patients to engage in efforts to alleviate it. One key mechanism is meaning-making, which involves deliberate cognitive activities such as interpreting one’s situation, considering potential coping strategies, and organizing daily life to reduce emotional discomfort [17,20]. Meaning-making aims to restore disrupted psychological balance by fostering intrapsychic approaches that generate new, acceptable ways of understanding the situation. When meaning-making is successful and constructive, patients can view their illness and its consequences differently, without undermining or violating their core beliefs and personal goals [17]. Consequently, this is associated with lower intensity of illness-specific fears, such as fear of recurrence, as well as reduced broader emotional distress, including generalized anxiety. Conversely, in cases of maladaptive meaning-making, greater negative changes occur in beliefs and goals, which consequently are related to higher levels of fear and anxiety.

Second, the identified dual pathway through which illness perceptions exert their influence involves negative perceptions increasing vulnerability to both illness-specific fear and generalized anxiety by disrupting meaning-making and altering beliefs and goals. In contrast, positive perceptions reduce fear and anxiety by promoting adaptive meaning-making and stability in beliefs and goals. These two complementary routes—maladaptive and adaptive—are also supported by previous research on coping with traumatic events and illness [16,21]. Specifically, negative illness perceptions in the form of viewing the illness as highly threatening, uncontrollable, or unpredictable appear to undermine adaptive cognitive processing by disrupting meaning-making. This disruption is associated with greater changes in beliefs and goals, reflecting motivational instability, which heightens vulnerability to fear of recurrence as well as generalized anxiety [29]. Conversely, positive illness perceptions, such as perceiving the illness as manageable or having a personal value, facilitate constructive meaning-making and promote stability in beliefs and goals. By enabling patients to integrate the illness experience into a coherent cognitive and motivational framework, these perceptions reduce both illness-specific fears and broader anxiety.

This interpretation is consistent with the meaning-making theory proposed by Park [15,25], which emphasizes that adaptive reappraisal and the restoration of coherence between situational appraisals and global beliefs can buffer against psychological distress. In contrast, maladaptive processes are undeniably associated with increased negative emotional states. Moreover, this distinction is supported by previous research, which has shown that cancer—depending on an individual’s capacity for constructive and meaningful thinking—can be reinterpreted as, naturally, adverse and traumatic, but also as a potential opportunity for development or post-traumatic growth in women with breast cancer [44] and different cancer survivors [42]. This is attributable to the inherent nature of cancer as a chronic illness, which is associated with a significant emotional burden, particularly in relation to the projected course of the disease and the uncertainty surrounding the future.

### 4.3. Clinical Implications

The findings of the present study, while primarily presenting empirical data and their interpretations, also provide a substantive foundation for deriving clinical implications relevant to post-treatment cancer patients, their families, and multidisciplinary treatment teams, including psychologists, physicians, and nurses. Emotional reactions and psychological adjustment in cancer patients can be facilitated by meaning-based therapeutic interventions that go beyond the provision of procedural or medical information, encompassing the individual’s meaning-making system and the unique challenges of confronting cancer [19,22]. Furthermore, a coherent system of meanings, grounded in personal beliefs and goals, may serve a protective and adaptive function and facilitate reflective engagement with the existential dimensions of one’s life; hence, interventions aimed at enhancing patients’ meaning in life are warranted [30]. Potential clinical advantages may also include helping patients who have completed cancer treatment recognize that successful adaptation to illness depends not only on the effectiveness of medical interventions, but also on their perceptions of the illness and their understanding of themselves within the broader context of personal beliefs and goals.

For post-treatment thoracic cancer patients, interventions may also be tailored using meaning-centered or cognitive–motivational approaches, depending on whether the primary source of distress is fear of cancer recurrence or generalized anxiety. Patients with elevated fear or recurrence may benefit particularly from: (1) meaning-centered interventions that help reframe cancer-related experiences and facilitate the development of concrete long-term goals, and (2) cognitive–motivational techniques that enable modification of maladaptive thought patterns, rendering them less threatening to current health. In contrast, patients with high generalized anxiety may require broader interventions focusing on worry management, uncertainty tolerance, and reflection on life goals. Furthermore, combining meaning-centered approaches with cognitive–behavioral skills can simultaneously address existential concerns and maladaptive cognitive patterns, ultimately enhancing psychological resilience. These approaches align with our mediation model, suggesting that fostering meaning-making and adaptive changes in beliefs and goals may reduce both fear and anxiety in post-treatment thoracic cancer patients. This leads to a specific clinical recommendation to incorporate aspects directly related to goals, values, and meaning into patient care.

### 4.4. Limitations

Several limitations of the current study should be acknowledged. First, our study was limited to the use of specific instruments measuring illness perception and changes in beliefs and goals at a global level. Although both questionnaires demonstrate good psychometric properties and yielded interesting results, future research should employ tools that more precisely assess illness perceptions and changes in beliefs and goals among cancer patients (e.g., assessing specific life domains or spheres of personal interest). Second, the design and analytical approach of the study were cross-sectional, which precludes causal inferences. Although the model was grounded in an established theoretical framework—the meaning-making theory [16,24], longitudinal studies are required to determine whether the end of treatment predicts changes in fear of recurrence and anxiety. Finally, the inclusion criteria applied in the current study—specifically, requiring patients to be in remission, excluding stage IV disease, and including only those with a positive treatment response—limit the generalizability of our findings. Therefore, the results primarily apply to post-treatment thoracic cancer patients who have achieved remission and are clinically stable. Caution should be exercised when extrapolating these findings to patients with advanced or metastatic disease, those currently undergoing active treatment, or individuals with significant comorbidities, as their illness perception, fear of recurrence, and psychological adjustment may differ substantially.

## 5. Conclusions

This study provided an in-depth examination of the mediating role of cognitive and motivational factors in regulating emotional reactions among patients with thoracic cancer after treatment. The main findings demonstrated a serial and parallel mediation effect of meaning-making and changes in beliefs and goals between illness perception, fear of recurrence, and generalized anxiety. In light of the meaning-making model [15,25], this contributes to a more nuanced understanding of how changes within a broad meaning system—encompassing core beliefs, life goals, and personal values—shape post-treatment fear and anxiety. Additionally, the results underscore the importance of meaning reconstruction in shaping emotional responses to the cancer experience, highlighting how changes in patients’ interpretative frameworks may influence the relationship of illness perception with fear and anxiety associated with illness-related dangers. In this context, psychological and medical resources should be allocated to the development of meaning-based interventions to support post-treatment cancer patients, and healthcare professionals should be trained in the psychosocial aspects of cancer care.

## Figures and Tables

**Table 1:** Correlations among illness perception, meaning-making, changes in beliefs and goals, fear of recurrence, and generalized anxiety.

	*M*	*SD*	1.	2.	3.	4.	5.	6.	7.
1. Age	51.33	12.70	–						
2. Negative illness perception	3.60	0.88	0.21 ***	–					
3. Positive illness perception	3.08	0.76	0.05	−0.20 **	–				
4. Meaning-making	2.93	1.14	−0.18 **	−0.23 ***	0.45 ***	–			
5. Changes in beliefs	5.50	1.22	0.24 ***	0.32 ***	−0.15 *	−0.38 ***	–		
6. Changes in goals	5.30	0.90	−0.03	0.23 ***	−0.41 ***	−0.41 ***	0.52 ***	–	
7. Fear of recurrence	2.91	0.51	0.09	0.19 **	−0.40 ***	−0.48 ***	0.40 ***	0.48 ***	–
8. Generalized anxiety	1.59	0.65	0.14 *	0.42 ***	−0.22 ***	−0.33 ***	0.43 ***	0.40 ***	0.48 ***

* *p* < 0.05; ** *p* < 0.01; *** *p* < 0.001.

**Table 2:** Hierarchical regression results for gender, age, illness perception, meaning-making, and changes in beliefs and goals as predictors of fear of recurrence and generalized anxiety, respectively.

Variables	R^2^	ΔR^2^	β	t
Fear of recurrence				
Model 1	0.01			
Gender			−0.01	−0.08
Age			0.09	1.51
Model 2	0.20	0.19 ***		
Gender			−0.05	−0.91
Age			0.09	1.62
Negative illness perception			0.10	1.74
Positive illness perception			−0.41	−7.26 ***
Model 3	0.38	0.19 ***		
Gender			−0.06	−0.91
Age			0.01	1.62
Negative illness perception			0.05	0.96
Positive illness perception			−0.20	−7.26 ***
Meaning-making			−0.48	−8.75 ***
Model 4	0.43 (adjusted R^2^)	0.06 **		
Gender			−0.05	−0.91
Age			0.01	0.04
Negative illness perception			−0.01	−0.16
Positive illness perception			−0.15	−2.67 ***
Meaning-making			−0.39	−8.75 ***
Changes in beliefs			0.13	2.19 *
Changes in goals			0.20	3.28 ***
Gender			−0.05	−0.91
Generalized anxiety				
Model 1	0.02			
Gender			0.02	0.77
Age			0.14	2.34 *
Model 2	0.21	0.19 ***		
Gender			−0.04	−0.76
Age			0.07	1.22
Negative illness perception			0.38	6.71 ***
Positive illness perception			−0.16	−2.84 **
Model 3	0.25	0.05 **		
Gender			−0.05	−0.85
Age			0.03	0.47
Negative illness perception			0.35	6.38 ***
Positive illness perception			−0.06	−0.98
Meaning-making			−0.23	−3.70 ***
Model 4	0.32 (adjusted R^2^)	0.08 ***		
Gender			−0.03	−0.60
Age			0.02	0.36
Negative illness perception			0.28	5.23 ***
Positive illness perception			−0.02	−0.27
Meaning-making			−0.14	−1.97 *
Changes in beliefs			0.19	2.93 **
Changes in goals			0.19	2.84 **

* *p* < 0.05; ** *p* < 0.01; *** *p* < 0.001.

**Table 3:** Mediational effects based on bootstrapped standardized estimates and 95% confidence intervals for the final mediation model.

Model Pathways	Estimate	95% CI	
		Lower	Upper
Model with serial and parallel mediation effects			
NIP → M-M (mediator 1) → ChB/ChG (mediators 2) → FoR	0.10 ^a^	0.04	0.16
NIP → M-M (mediator 1) → ChB/ChG (mediators 2) → GA	0.07 ^a^	0.04	0.10
PIP → M-M (mediator 1) → ChB/ChG (mediators 2) → FoR	−0.25 ^a^	−0.31	−0.08
PIP → M-M (mediator 1) → ChB/ChG (mediators 2) → GA	−0.13 ^a^	−0.18	−0.11
M-M → ChB/ChG (parallel mediators) → FoR	−0.11 ^a^	−0.17	−0.09
M-M → ChB/ChG (parallel mediators) → GA	−0.14 ^a^	−0.14	−0.20
NIP → M-M (single mediator) → ChB	0.05 ^a^	0.02	0.10
NIP → M-M (single mediator) → ChG	0.06 ^a^	0.02	0.09
PIP → M-M (single mediator) → ChB	−0.14 ^a^	−0.19	−0.10
PIP → M-M (single mediator) → ChG	−0.12 ^a^	−0.17	−0.08
NIP → M-M (single mediator) → FoR	0.05 ^a^	0.02	0.06
PI → M-M (single mediator) → FoR	−0.12 ^a^	−0.13	−0.01

^a^ Empirical 95% confidence interval does not overlap with zero. Abbreviations: Negative illness perception—NIP; Positive illness perception—PIP; Meaning-making—M-M; Changes in beliefs—ChB; Changes in goals—ChG; Fear of recurrence—FoR; Generalized anxiety—GA.

## Data Availability

The data presented in this study are available at URL: https://osf.io/wrnm8/files/osfstorage (accessed on 15 January 2026).
